# The Effects of Chatbot Recovery Strategies and Anthropomorphic Image Type on Service Recovery Satisfaction: The Mediating Roles of Perceived Warmth and Competence

**DOI:** 10.3390/bs16091564

**Published:** 2026-09-03

**Authors:** Ye Wang, Yaoqun Xu, Tung-Ju Wu

**Affiliations:** 1 School of Computer and Information Engineering, Harbin University of Commerce, Harbin 150028, China; wangye@s.hrbcu.edu.cn; 2 School of Management, Harbin Institute of Technology, Harbin 150001, China

**Keywords:** chatbot recovery, recovery strategy, anthropomorphic image, service failure, service recovery satisfaction, perceived warmth, perceived competence

## Abstract

The widespread adoption of chatbots enables companies to provide customer service at low cost and with high efficiency. However, the inherent limitations of chatbots often lead to service failures, highlighting the necessity of effective recovery strategies. Nevertheless, the optimal approaches for chatbots to implement self-recovery strategies remain unclear. Drawing on the Computers As Social Actors (CASA) paradigm and the Stereotype Content Model (SCM), this study investigates the interactive effects of recovery strategies (emotional recovery strategy vs. informational recovery strategy) and anthropomorphic image types (warm image vs. competent image) on service recovery satisfaction. It also examines the psychological mechanisms and boundary conditions underlying these effects. Based on two offline experiments with university students (Study 1: *n* = 162; Study 2: *n* = 350), the results show that a match between a chatbot’s emotional recovery strategy and a warm image (or an informational recovery strategy and a competent image) enhances service recovery satisfaction. Perceived warmth and perceived competence are found to mediate these effects. The findings further reveal that time pressure moderates the interactive effect. These findings advance the theoretical understanding of the psychological mechanisms underlying the effects of chatbot self-recovery strategies on service recovery satisfaction, while offering practical implications for optimizing service recovery strategies and enhancing customer experience.

## 1. Introduction

Advances in artificial intelligence (AI) and natural language processing have driven the widespread adoption of chatbots across the retail and service sectors. Chatbots are autonomous computer programs that enable real-time interactions with users through natural language (Mozafari et al., 2022; Sands et al., 2021). Owing to their 24/7 availability, rapid response, and cost efficiency, chatbots have been widely adopted in customer service, and an increasing number of firms are replacing human employees with chatbots to reduce costs and improve service efficiency (Ashfaq et al., 2020; Bagherzadeh et al., 2020; Brendel et al., 2023; H. Meng et al., 2025a). Although the intelligence of chatbots continues to improve, service failures remain inevitable due to inherent issues such as response delays or mismatched answers, as well as the complexity of service environments and the diversity of customer needs (Pavone et al., 2023; Xing et al., 2022). These service failures may result in reduced consumer trust, anger, and dissatisfaction, and further result in negative word-of-mouth, brand avoidance behaviors, and reduced intentions to continue using chatbots (Jin, 2026; Mou et al., 2026; Ho et al., 2026; Castillo et al., 2021; Kuanr et al., 2025; Song et al., 2022). Existing studies have examined the differences between chatbot self-recovery and human recovery (Lu & Min, 2025). Therefore, how to effectively reduce the negative impacts of chatbot service failures through self-recovery strategies and enhance consumers’ service recovery satisfaction has become an important research issue in intelligent service contexts.

Prior research in the service recovery domain suggests that a highly anthropomorphic chatbot image can facilitate recovery outcomes by enhancing consumers’ willingness to forgive (Agnihotri & Bhattacharya, 2024; H. Liu et al., 2026). However, other studies have shown that anthropomorphic chatbot cues may generate unfavorable consumer responses in service failure contexts, such as reduced purchase intentions (Esmark Jones et al., 2026), and that competent chatbot images may even exacerbate consumers’ disappointment. These inconsistent findings indicate that anthropomorphism does not necessarily generate positive outcomes in service recovery contexts. Rather, its effectiveness may depend not only on the level of anthropomorphism but also on the specific social attributes conveyed by anthropomorphic images. Drawing on the Stereotype Content Model (SCM) (Cuddy et al., 2008), prior research has distinguished chatbot anthropomorphic images into warm images and competent images (Q. Yao et al., 2023). However, prior research has mainly examined how different types of anthropomorphic images independently influence consumer responses, leaving the conditions under which these images shape consumer evaluations during service recovery largely unexplored. According to the Computers As Social Actors (CASA) paradigm, individuals transfer social norms from human-to-human interactions to their interactions with chatbots (Nass et al., 1994; Reeves & Nass, 1996) and make corresponding judgments based on the social cues conveyed by chatbots. Recovery strategies serve as an important means through which chatbots convey social information. Prior research has commonly classified chatbot self-recovery strategies into emotional recovery strategies (emphasizing emotional support) and informational recovery strategies (emphasizing information provision and problem-solving) (Zhou & Chang, 2024). Accordingly, recovery strategies, as important linguistic cues, may interact with anthropomorphic images as visual cues to shape consumers’ service recovery evaluations. This study adopts a social cue integration perspective to investigate the interaction between recovery strategies and anthropomorphic image types. Specifically, we examine how linguistic cues and visual cues jointly shape consumers’ social cognitive judgments of chatbots and account for the differential effects of anthropomorphic cues in service recovery contexts, thereby offering new insights into enhancing the effectiveness of chatbot self-recovery. Furthermore, this study explores how this interaction effect influences service recovery satisfaction through two core social perception dimensions: perceived warmth and perceived competence.

Time pressure is a critical situational factor affecting chatbot service recovery outcomes (Lv et al., 2021; Xu & Liu, 2022). Recent research has begun to investigate how time pressure shapes chatbot service recovery effectiveness, with existing studies primarily focusing on emotional recovery strategies. Under conditions of low time pressure, previous studies have shown that appreciation is more effective than apology in enhancing service recovery satisfaction (Song et al., 2023), empathetic responses can improve customer experience (Juquelier et al., 2025), and symbolic recovery can facilitate customer forgiveness (Zaki & Al-Romeedy, 2024). However, under high time pressure conditions, the beneficial effects of these strategies may be attenuated. Specifically, the differential effects of politeness strategies (appreciation vs. apology) disappear (Song et al., 2023), empathetic cues may even produce negative effects (Juquelier et al., 2025), and the effectiveness of symbolic recovery is reduced (Zaki & Al-Romeedy, 2024). Importantly, chatbot service recovery involves not only emotional responses but also informational support aimed at resolving service problems, namely, informational recovery strategies. Prior research has mainly examined the effects of time pressure on emotional recovery strategies, while paying limited attention to how time pressure changes the relative effectiveness of different recovery strategies. When emotional recovery strategies become less effective under high time pressure, it remains unclear whether informational recovery strategies may gain a relative advantage and how this contextual factor further shapes the effectiveness of the match between recovery strategies and anthropomorphic image types. Therefore, this study introduces time pressure as a boundary condition and investigates how it moderates the effect of congruence between recovery strategies and anthropomorphic image types on service recovery satisfaction.

Therefore, this study seeks to address these research gaps by answering the following two research questions:

RQ1: How does the interaction between chatbot recovery strategies (emotional recovery strategy vs. informational recovery strategy) and anthropomorphic image types (warm image vs. competent image) influence consumers’ service recovery satisfaction? What are the underlying psychological mechanisms?

RQ2: How does time pressure moderate this process?

Drawing on the CASA paradigm and SCM, this study develops a theoretical model to address the aforementioned research questions and contributes to the chatbot service recovery literature in the following ways. First, this study extends the application of the CASA paradigm in chatbot service recovery contexts by demonstrating how multiple social cues jointly shape consumers’ evaluations of chatbot service recovery. While prior research has primarily examined the effects of individual social cues on consumer responses, this study reveals the interactive effects between recovery strategies and anthropomorphic image types. This study advances the understanding of how the CASA paradigm operates through the integration of multidimensional social cues in chatbot interactions and provides a theoretical explanation for the heterogeneous effects of anthropomorphism in service recovery research. Drawing on SCM, this study further uncovers the social cognitive mechanisms underlying this interaction by demonstrating how perceived warmth and perceived competence mediate its effects on service recovery satisfaction. Second, this study identifies the moderating role of time pressure in the congruence effect between recovery strategies and anthropomorphic image types, extending the understanding of how time pressure functions as a contextual factor in chatbot service recovery. While prior research has mainly examined the effects of time pressure on emotional recovery strategies, this study advances existing knowledge by investigating how time pressure shapes the relative effectiveness of different recovery strategies and alters the effectiveness of their congruence with anthropomorphic image types. These findings provide theoretical insights into the boundary conditions under which chatbot recovery strategies are effective in dynamic service environments. Furthermore, this study offers practical implications for firms seeking to optimize the design and implementation of chatbot service recovery strategies.

## 2. Theoretical Basis and Research Hypothesis

### 2.1. Effects of the Interaction of Recovery Strategies and Anthropomorphic Image Types on Recovery Satisfaction

Based on the CASA paradigm, individuals tend to unconsciously apply the same social rules used in human–human interactions when interacting with computers, thereby eliciting social responses and perceiving computers as social actors (Nass et al., 1994; Reeves & Nass, 1996). Such social responses are primarily triggered by social cues presented by computers. Users form social cognitions based on cues such as language, visual appearance, behavior, and voice, and subsequently generate corresponding interactive responses (Ham et al., 2024).

In chatbot interaction contexts, social cues are mainly conveyed through linguistic and visual cues (Y. Huang et al., 2021; Agnihotri & Bhattacharya, 2024). Linguistic cues, including communication styles (S. Wang et al., 2023), emotional expressions, response strategies (Haupt et al., 2023), and other related forms, convey social information about chatbots and shape users’ perceptions of their social attributes, such as interaction intentions and emotional characteristics in social interactions. Visual cues, including anthropomorphic images (Lu et al., 2024), levels of anthropomorphism (M. Nguyen et al., 2023), and other related visual cues, provide social information that shapes users’ evaluations of chatbots’ social attributes and personality traits (M. Kim, 2024). In the service recovery context of this study, linguistic cues are primarily presented through the recovery strategies adopted by chatbots. Existing research has shown that service providers typically employ different types of recovery strategies when service failures occur, among which emotional recovery strategies and informational recovery strategies are two important forms of remediation (Zhou & Chang, 2024).

The emotional recovery strategy focuses on addressing consumers’ affective needs following service failure by offering empathy, understanding, and comfort. It communicates care and concern through apologies, acknowledgment of consumers’ feelings, and the provision of emotional support. Within the Computers As Social Actors (CASA) paradigm, consumers are likely to interpret such emotionally supportive responses from chatbots as social behaviors akin to human interpersonal interaction, which in turn increases their perception that the service agent is attentive to their experience. Accordingly, emotional recovery strategies help mitigate consumers’ negative emotions triggered by service failure and foster more favorable evaluations of the recovery process (R. Huang & Ha, 2020). The informational recovery strategy focuses on resolving service issues by addressing consumers’ informational needs, specifically through offering explanations of failure causes, delivering clear and accurate information, and providing relevant solutions (Wirtz et al., 2018). Within the Computers As Social Actors (CASA) paradigm, consumers tend to infer chatbots’ competence and service value from the informational cues they exhibit, leading to the perception that chatbots have both the intention and capability to resolve problems. Within chatbot service recovery interactions, strategies that proactively offer explanations or alternative answer options are more effective in fostering user acceptance and shaping recovery strategy preferences (Ashktorab et al., 2019). Therefore, informational recovery strategies can reduce consumers’ uncertainty regarding service failures and enhance their evaluation of recovery effectiveness.

However, consumers’ evaluations of chatbot service recovery effectiveness are not determined solely by linguistic cues. Drawing on the CASA paradigm, consumers tend to integrate multiple social cues presented by chatbots to form an overall judgment. Accordingly, the fit between recovery strategies and the linguistic and visual cues embedded in anthropomorphic images further shapes consumers’ evaluations of service recovery outcomes. When chatbots are designed with anthropomorphic images, this not only enhances users’ sense of social presence and closeness but also facilitates a more intuitive understanding of system functions, thereby enabling the recovery strategy to be more effectively realized (Go & Sundar, 2019). According to the SCM and subsequent research, consumers’ perceptions of social agents are primarily structured along two fundamental dimensions: warmth and competence (Fiske et al., 2007; Judd et al., 2005; Cuddy et al., 2008). Consistent with face perception research showing that visual cues can rapidly elicit trait inferences (Todorov et al., 2005; Willis & Todorov, 2006), different anthropomorphic images can convey distinct social cues. Accordingly, chatbot anthropomorphic images can be differentiated into warm and competent images (Q. Yao et al., 2023).

A warm image is typically conveyed through cues such as a broad smile or casual attire, which tend to be associated with higher perceived warmth (Hertenstein et al., 2009; Zheng et al., 2022). These visual signals are more likely to elicit consumers’ emotional responses and perceptions of interpersonal care, thereby more effectively fulfilling their emotional needs (Z. Wang et al., 2017). Emotional recovery strategies emphasize empathy, understanding, and emotional support in responding to consumer needs, and the conveyed sense of care is consistent with the social signals embedded in a warm anthropomorphic image. Accordingly, the congruence between emotional recovery strategies and a warm anthropomorphic image can enhance consumers’ positive evaluations of the recovery process, thereby increasing service recovery satisfaction. By contrast, a competent image is typically signaled through cues such as a slight smile or formal professional attire (Eaves & Leathers, 2017; Kraus & Chen, 2013). Such cues are generally associated with higher perceived competence but lower perceived warmth, while simultaneously triggering consumers’ perceptions of competence and professionalism (Cuddy et al., 2007). The informational recovery strategy focuses on addressing consumer needs through providing causal explanations, offering solutions, and demonstrating problem-solving capability. Its emphasis on effectiveness and professionalism is more congruent with the attributes conveyed by a competent image. Accordingly, the congruence between the informational recovery strategy and a competent anthropomorphic image can further enhance consumers’ favorable evaluations of recovery effectiveness, thereby increasing service recovery satisfaction.

Therefore, based on the Computers As Social Actors (CASA) paradigm, consumers cognitively process both linguistic and visual social cues presented by chatbots in an integrated manner. When the information conveyed by the recovery strategy is consistent with that conveyed by the anthropomorphic image, consumers are more likely to form positive social judgments and enhance their service recovery satisfaction. The match between a chatbot’s recovery strategy and its anthropomorphic image can lead to more effective recovery outcomes. When the chatbot presents a warm image, the emotional recovery strategy more effectively improves service recovery satisfaction; whereas when the chatbot presents a competent image, the informational recovery strategy leads to higher satisfaction. Accordingly, we propose the following hypotheses.

**H1.**  *There is an interaction effect between chatbot recovery strategies and anthropomorphic images on consumers’ service recovery satisfaction.*

**H1a.**  *When a warm anthropomorphic image is matched with an emotional recovery strategy (compared with an informational recovery strategy), it leads to higher customer service recovery satisfaction.*

**H1b.**  *When a competent anthropomorphic image is matched with an informational recovery strategy (compared with an emotional recovery strategy), it leads to higher customer service recovery satisfaction.*

### 2.2. The Mediating Role of Perceived Warmth and Perceived Competence

Drawing on the SCM, individuals tend to rely on stereotypes as cognitive heuristics when cognitive resources are limited, thereby reducing cognitive load and enhancing judgment efficiency (El Hedhli et al., 2023). Within this framework, SCM identifies two fundamental dimensions: warmth and competence. Warmth is associated with traits such as kindness, friendliness, and understanding (Haupt et al., 2023), whereas competence is linked to skillfulness, capability, and efficiency (Cai et al., 2024; R. Yao et al., 2024). These two dimensions reflect individuals’ social perceptions and evaluations of social targets, through which individuals infer the intentions and capabilities of social targets based on available social information. In chatbot service recovery contexts, consumers’ perceptions of chatbot warmth and competence are formed through the integration and interpretation of multiple social cues, including linguistic cues conveyed by recovery strategies and visual cues embedded in anthropomorphic images. These social evaluations subsequently influence consumers’ satisfaction with service recovery outcomes.

During service recovery processes, consumers are concerned not only with whether the problem has been resolved, but also with whether the chatbot demonstrates care and empathy (Lidén & Skålén, 2003). In chatbot interaction contexts, although chatbots do not possess genuine emotions, they can still convey emotional support to consumers through linguistic expressions and interaction styles (J. Meng & Dai, 2021). Emotional recovery strategies provide warmth-related linguistic cues by expressing apologies, demonstrating understanding, and providing reassurance (Wirtz et al., 2018). Meanwhile, a warm anthropomorphic image provides visual cues associated with kindness and care, such as a broad smile and casual attire (Z. Wang et al., 2017; Q. Yao et al., 2023). However, consumers’ warmth judgments are not determined by any single cue; rather, they emerge from the integration and interpretation of multiple social signals. When these two cues are combined, they jointly strengthen consumers’ warmth-related social judgments toward the chatbot, allowing consumers to infer the chatbot’s caring intentions from converging warmth-related cues and form stronger judgments regarding its benevolence and care than when either cue is presented alone. This enhanced perception of warmth subsequently shapes consumers’ evaluations of the recovery outcome. Prior research in service robotics has also demonstrated that perceived warmth can enhance satisfaction after service failures (Choi et al., 2021). Accordingly, the following hypothesis is proposed.

**H2.**  *Perceived warmth mediates the positive effect of a chatbot’s emotional recovery strategy and warm image on service recovery satisfaction.*

In the context of service recovery, consumers primarily assess whether chatbots are capable of effectively solving problems. Chatbots’ informational recovery strategy provides competence-related linguistic cues that strengthen consumers’ perceptions of chatbot competence by diagnosing issues, explaining the underlying causes of service failures, and providing appropriate solutions (Marinova et al., 2018). More specifically, chatbots can identify potential problems and clarify the reasons behind service failures, thereby enabling consumers to better understand the situation and perceive the chatbot as capable of handling the problem effectively. Moreover, the provision of relevant solutions further strengthens consumers’ evaluations of chatbot competence (Xu & Liu, 2022). Meanwhile, a competent anthropomorphic image provides visual cues associated with professionalism and capability, such as formal professional attire and a confident appearance (Q. Yao et al., 2023). However, consumers’ competence judgments are not determined by any single cue; rather, they emerge from the interpretation of multiple social signals. When these two cues are combined, they jointly strengthen consumers’ competence-related social judgments toward the chatbot, allowing consumers to integrate consistent capability-related signals and form stronger perceptions of the chatbot’s competence and problem-solving ability than when either cue is presented alone. This enhanced perception of competence reflects consumers’ evaluations of the chatbot’s professionalism, effectiveness, and service reliability. When consumers perceive that the chatbot possesses strong problem-solving capabilities, they are more likely to trust its ability to handle service failures and evaluate the recovery outcome more favorably, thereby increasing service recovery satisfaction (Haupt et al., 2023). Therefore, the following hypothesis is proposed.

**H3.**  *Perceived competence mediates the positive effect of a chatbot’s informational recovery strategy and competent image on service recovery satisfaction.*

### 2.3. Moderating Effect of Time Pressure

Compared with traditional interpersonal services, chatbots are increasingly deployed in time-sensitive service contexts where efficiency and rapid responses are highly valued (Juquelier et al., 2025), such as flight reservations, order processing, and urgent inquiries. Consumers often turn to chatbots expecting timely responses and efficient problem resolution. However, service failures not only create negative experiences by leaving consumers’ problems unresolved but also impose additional time constraints as consumers attempt to accomplish their original goals within a limited period, thereby increasing perceived time pressure (Song et al., 2023). Prior research has demonstrated that when facing time constraints, consumers interacting with chatbots have stronger expectations for timely and effective service recovery (Juquelier et al., 2025), and recovery timeliness significantly shapes consumer responses (Zaki & Al-Romeedy, 2024). Accordingly, in chatbot service failure contexts, time pressure serves as a critical situational factor that influences consumers’ evaluations of service recovery.

Drawing on the CASA paradigm, consumers’ responses to social cues provided by chatbots are largely automatic and heuristic in nature; however, such responses are contingent on the cognitive resources available to the individual. Time pressure, as a situational factor influencing the allocation of cognitive resources, refers to the perceived insufficiency of available time to complete a task, which gives rise to feelings of urgency or concern (Godinho et al., 2016). Under time constraints, consumers have limited cognitive resources available for information processing, which may alter how they process and prioritize social cues conveyed by chatbots. Accordingly, in service recovery contexts, time pressure not only shapes consumers’ information processing but also influences their attention to and reliance on different types of social cues, thereby affecting the effectiveness of various recovery strategy and anthropomorphic image combinations.

Under high time pressure, consumers’ cognitive resources available for information processing are limited, making it difficult for them to process different types of information conveyed by chatbots fully. They place greater emphasis on efficiency and tend to prefer clear, certain, and task-relevant information that enables them to resolve problems quickly (Q. N. Nguyen et al., 2022). Accordingly, they are more concerned with whether chatbots can effectively address service problems and assist them in achieving their task objectives (Song et al., 2023). Specifically, informational recovery strategy provides consumers with clear directions for problem resolution by explaining the causes of service failures, describing corrective actions, and offering solutions (Wirtz et al., 2018). A competent image enhances consumers’ perceptions of chatbot recovery capability by providing visual cues related to professionalism and reliability. When an informational recovery strategy is congruent with a competent image, the consistent signals conveyed by these two cues enable consumers to rapidly evaluate recovery effectiveness. By contrast, although an emotional recovery strategy and a warm image convey signals of care and emotional support, these warmth-related cues are less relevant to consumers’ task-oriented needs under high time pressure, thereby exerting a weaker influence on recovery evaluations (Juquelier et al., 2025). Therefore, under high time pressure conditions, the congruence between an informational recovery strategy and a competent image is more likely to enhance consumers’ service recovery satisfaction.

**H4.**  *Under high time pressure, a competent image combined with an informational recovery strategy (compared with a warm image and an emotional recovery strategy) leads to higher service recovery satisfaction.*

When consumers experience low time pressure, they possess sufficient cognitive resources for information processing, enabling them to comprehensively interpret different types of social cues conveyed by chatbots. Under these conditions, consumers’ evaluations of chatbot recovery capability and service performance are not limited to whether the service problem has been resolved; rather, they also place greater emphasis on the emotional experience generated during the interaction (Y. S. S. Huang & Dootson, 2022). Additionally, emotional recovery behaviors, such as apologies and expressions of gratitude, can promote consumers’ positive evaluations of recovery outcomes (Zaki & Al-Romeedy, 2024; Song et al., 2023). Specifically, an emotional recovery strategy provides emotional support by expressing apologies, demonstrating understanding, and offering reassurance, while a warm image enhances consumers’ perceptions of the chatbot’s positive interaction intentions through friendly and approachable visual cues. When an emotional recovery strategy is congruent with a warm image, consumers receive consistent emotional cues from both linguistic and visual dimensions, thereby enhancing their evaluations of service recovery (Zaki & Al-Romeedy, 2024). In contrast, an informational recovery strategy and a competent image primarily provide information related to professionalism and problem-solving. Although such task-oriented information can also contribute to consumers’ positive evaluations, consumers under low time pressure have sufficient cognitive resources to process various types of information during the interaction. Consequently, emotion-related information plays a more important role in shaping recovery evaluations. Therefore, under conditions of low time pressure, when a warm image is aligned with an emotional strategy (as opposed to a competent image combined with an informational strategy), it can significantly improve service recovery satisfaction.

**H5.**  *Under low time pressure, the combination of a warm image and an emotional strategy (compared with a competent image and an informational strategy) leads to higher customer service recovery satisfaction.*

**Figure 1:**
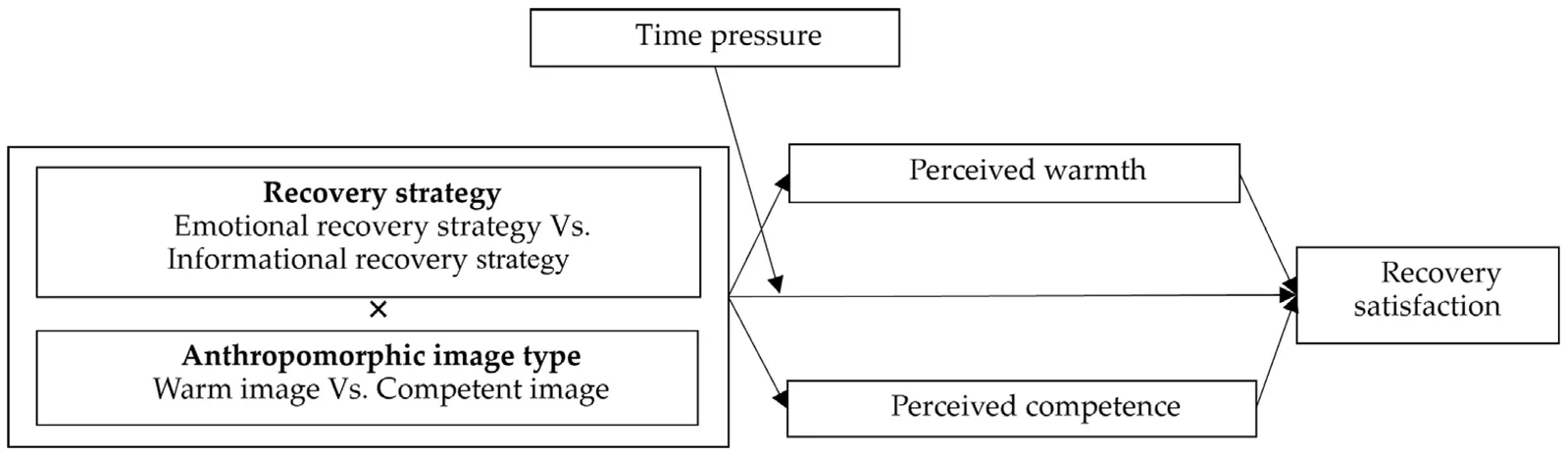
Research model.

Based on the above hypothesis, the theoretical research model of this study is shown in Figure 1.

## 3. Method

This study employed a scenario-based experiment and collected data in a laboratory setting. Scenario-based experiments enable researchers to manipulate key variables within experimental scenarios and examine participants’ responses across different conditions, thereby facilitating the identification of causal relationships and improving research validity (Qin & Li, 2020). Experimental materials combining text and images were presented to participants to facilitate scenario immersion, after which they completed the questionnaire.

Both studies conducted pretests prior to the formal experiments to validate the effectiveness of the experimental materials. Study 1 aimed to examine the interaction effects of recovery strategies and anthropomorphic image types on service recovery satisfaction (H1, H1a, and H1b) and further tested the mediating effects of perceived warmth and competence (H2 and H3). Study 2 further examined the moderating role of time pressure in these interaction effects (H4 and H5).

### 3.1. Study 1: Interactive Effects of Recovery Strategies and Anthropomorphic Types on Recovery Satisfaction

Study 1 investigates the interactive effects of recovery strategies (emotional recovery strategy vs. informational recovery strategy) and anthropomorphic image (warm image vs. competent image) on customer recovery satisfaction, while also examining the mediating roles of perceived warmth and perceived competence. All participants were recruited from a university in Northeast China.

#### 3.1.1. Pretest

Pretest 1a was conducted to examine the effectiveness of the chatbot recovery strategies (emotional recovery strategy vs. informational recovery strategy). The experimental scenarios were developed based on prior research. Specifically, the experimental scenarios involved service failures occurring during the service delivery process (Smith et al., 1999). Chatbot service failures are commonly manifested as response failures or dialogue interruptions, where chatbots fail to accurately identify users’ needs or provide effective responses (Haupt et al., 2023). Moreover, the overall scenario design was adapted from the framework developed by Zhou and Chang (2024) and further tailored to the context of chatbot service recovery. A total of 63 student participants participated in the pretest and were randomly assigned to either the emotional recovery strategy group or the informational recovery strategy group. Participants were then instructed to read the corresponding stimulus materials. In the emotional recovery condition, the scenario was presented as follows: “A consumer used a chatbot to book a flight from Harbin to Tianjin. After approximately 20 s of searching, the chatbot reported an unknown error and apologized. It also expressed empathy and understanding toward the consumer and encouraged the consumer to try again. The consumer subsequently provided more detailed booking information. After another 20 s of searching, the chatbot successfully completed the booking and wished the consumer a pleasant journey.”

For the informational recovery strategy condition, participants were presented with the following scenario: “A consumer used a chatbot to book a flight from Harbin to Tianjin. After approximately 20 s of search, the chatbot returned an unknown error while simultaneously providing possible causes and corresponding solutions. The consumer then resubmitted more detailed booking information and attempted again. The chatbot conducted another 20-s search and successfully completed the booking, presenting the complete flight information.” Subsequently, participants then evaluated whether the chatbot adopted an emotional recovery strategy or an informational recovery strategy using a 7-point Likert scale (1 = emotional recovery strategy, 7 = informational recovery strategy). An independent-samples *t*-test confirmed the effectiveness of the manipulation of chatbot recovery strategies. (M_emotion_ = 2.22, SD_emotion_ = 1.039; M_information_ = 5.65, SD_information_ = 0.989; *t* = −13.272, *p* < 0.001). These results confirm the success of the manipulation.

Pretest 1b was designed to assess whether the anthropomorphic image manipulation of the chatbot (warm image vs. competent image) was successfully perceived by participants. A sample of 66 student participants took part in the pretest and were randomly allocated to one of two experimental conditions: the warm image condition or the competent image condition. Participants were exposed to an avatar depicting either a warm image (a bright smile with casual attire) or a competent image (a slight smile with formal attire). Subsequently, participants evaluated their perceptions of both warm and competent attributes. The measurement items for warm image and competent image were adapted from Q. Yao et al. (2023), using a 7-point Likert scale (1 = strongly disagree; 7 = strongly agree). An independent-samples *t*-test revealed that participants in the warm image condition perceived the avatar as significantly warmer than competent (M_warm_ = 5.758, SD_warm_ = 1.206; M_competent_ = 2.453, SD_competent_ = 1.1242; *t* = 11.340, *p* < 0.001). Conversely, participants in the competent image condition perceived the avatar as significantly more competent than warm (M_competent_ = 5.956, SD_competent_ = 0.502; M_warm_ = 2.125, SD_warm_ = 0.414; *t* = −34.338, *p* < 0.001). These results confirm the success of the manipulation.

#### 3.1.2. Experimental Design and Procedure

This study recruited university students as experimental participants for several reasons. With the growing application of chatbots in online customer service and intelligent question-answering contexts, university students, as an important group of digital service users, are generally familiar with chatbot interaction styles and service processes. Given that chatbots may experience service failures due to issues such as information misinterpretation or insufficient problem recognition, university students are well-positioned to understand the experimentally designed service failure and recovery scenarios and to evaluate different recovery strategies accordingly. This study examines consumers’ psychological perceptions and behavioural responses in chatbot service recovery contexts, with a particular focus on the effects of recovery strategies and anthropomorphic image on service recovery satisfaction, rather than relying on specific industry experience or occupational background. Accordingly, university students, as general consumers with prior experience using chatbots, are well suited to understand the experimental scenarios and evaluate chatbot service performance. Prior research has demonstrated that the use of student samples does not undermine the external validity of research findings (Druckman et al., 2011). Moreover, this study adopts a controlled laboratory experimental design, in which key factors such as service failure scenarios, recovery strategies, and anthropomorphic image are systematically manipulated. This approach minimizes the influence of extraneous variables and allows for a more precise examination of the relationships among the focal constructs.

An a priori power analysis using G*Power 3.1.9 was conducted to determine the required sample size for the between-subjects design (Faul et al., 2007). Assuming an effect size of f = 0.25, an alpha level of 0.05, and a statistical power of 0.80, the analysis indicated that a minimum sample size of 128 participants was required. This study recruited 169 undergraduate students to participate in the experiment. After screening participants based on their prior chatbot usage experience, 166 eligible participants remained. Following data quality checks, four participants who failed the attention check were removed, yielding a final valid sample of 162 participants for subsequent analysis. The final sample size exceeded the minimum required sample size. Upon recruitment, participants were not informed of the specific research purpose or hypotheses. Participants were randomly assigned to one of the conditions in a 2 (recovery strategy: emotional recovery strategy vs. informational recovery strategy) × 2 (anthropomorphic image: warm image vs. competent image) between-subjects design. To minimize potential contamination from other manipulations, each participant was exposed to only one experimental condition, thereby reducing threats to internal validity. We provide detailed scenario descriptions in Appendix A.

Data collection was conducted through an offline experiment in March 2026 at a behavioral laboratory of a university located in Northeast China. The scenario experiment was initiated using a combination of text and images. The stimulus materials for chatbot recovery strategies and anthropomorphic images were identical to those used in the pretest. Prior to the formal experiment, participants were required to answer a screening question to confirm whether they had prior experience interacting with chatbots. Participants who answered “No” were excluded from the study, whereas those who answered “Yes” proceeded to the formal questionnaire phase (see Appendix D). Following the experimental design approach adopted in prior research (Y. Huang et al., 2021; Zhu et al., 2023), participants then read a scenario describing a service failure during the flight-booking process, followed by the chatbot’s service recovery response, and viewed the stimulus materials depicting the interaction between the customer and the chatbot (see Appendix A). They then completed the related questions. Subsequently, participants completed manipulation check items for anthropomorphic image types (warm image vs. competent image) and service recovery strategies (emotional recovery strategy vs. informational recovery strategy). Participants were then asked to assess their service recovery satisfaction, perceived warmth, perceived competence, as well as the perceived realism and immersion of the experimental scenario. To ensure the quality of the collected data, during the questionnaire survey, an attention check question was embedded in the questionnaire (Please select [Strongly Disagree] for this item). Respondents who selected any alternative option were classified as inattentive and were therefore excluded from the final dataset. Finally, participants reported their demographic information, including gender, age, education, monthly disposable allowance, and frequency of chatbot use. Each participant was compensated with 3 RMB for their participation.

#### 3.1.3. Preliminary Analysis

Manipulation check. A total of 166 responses were collected in this experiment. Four participants failed the attention check and were therefore excluded from the analysis, resulting in a final valid sample of 162 questionnaires. Among the participants, 63 were male (38.9%) and 99 were female (61.1%). The demographic characteristics of the participants are reported in Table 1.

An independent-samples *t*-test indicated that participants in the warm condition rated the warm image as significantly warmer than the competent image (M_warm_ = 5.905, SD_warm_ = 0.863; M_competent_ = 2.199, SD_competent_ = 0.489; *t* = 33.851, *p* < 0.001). Likewise, participants in the competent condition perceived the competent image as significantly more competent than the warm image (M_competent_ = 5.819, SD_competent_ = 0.576; M_warm_ = 1.652, SD_warm_ = 0.389; *t* = −53.725, *p* < 0.001), confirming successful manipulation of anthropomorphic image type. The manipulation check for chatbot recovery strategies also demonstrated significant differences between conditions (M_emotion_ = 2.11, SD_emotion_ = 1.107; M_information_ = 5.79, SD_information_ = 1.159; *t* = −20.663, *p* < 0.001), confirming successful manipulation. Furthermore, a one-sample *t*-test indicated that participants rated scenario realism at a relatively high level (M = 5.786, SD = 1.000; *t* = 73.638, *p* < 0.001). Scenario immersion was likewise rated highly (M = 5.776, SD = 0.966; *t* = 76.114, *p* < 0.001), suggesting that participants perceived the experimental setting as both realistic and immersive.

Assessment of measurement. Study 1 employed a seven-point Likert scale (1 = strongly disagree; 7 = strongly agree) to measure all constructs. All measurement items were adapted from existing validated scales and modified to fit the experimental context (see Appendix B).

Service recovery satisfaction was measured using a four-item scale adapted from Chung et al. (2020) (Cronbach’s α = 0.940). Perceived warmth was measured using a three-item scale adapted from Gao and Mattila (2014) (Cronbach’s α = 0.905). Perceived competence was measured using a three-item scale adapted from Cuddy et al. (2008) (Cronbach’s α = 0.828).

Scenario realism was measured following Crolic et al. (2022) (Cronbach’s α = 0.840). Scenario immersion was measured following Song et al. (2022) (Cronbach’s α = 0.770). Appendix C reports Cronbach’s α, composite reliability and average variance extracted for all constructs.

#### 3.1.4. Results

A total of 162 participants who passed the attention check were included in the final analysis. To reduce evaluation apprehension and self-report bias, all responses were collected anonymously throughout the experiment (Podsakoff et al., 2012). Harman’s single-factor test was conducted to assess common method bias (CMB). The results indicated that a single factor explained 35.454% of the total variance, less than the 50% baseline (Podsakoff et al., 2012). Furthermore, we adopted the unmeasured latent method construct (ULMC) approach by introducing a common latent factor into the CFA model. If the inclusion of the common latent factor does not result in a substantial improvement in model fit, common method bias (CMB) is unlikely to represent a serious concern (Podsakoff et al., 2012). Following conventional model fit criteria, CFI ≥ 0.95 and RMSEA ≤ 0.06 indicate good fit, whereas CFI ≥ 0.90 and RMSEA ≤ 0.08 indicate reasonable fit (Hu & Bentler, 1999). In study 1, the baseline CFA model demonstrated good fit (χ^2^/df = 1.329, CFI = 0.978, RMSEA = 0.045). After introducing the common latent factor (CLF), the model fit remained largely unchanged (χ^2^/df = 1.288, CFI = 0.984, RMSEA = 0.042), and the change in CFI (ΔCFI = 0.006) was below the 0.01 threshold (Cheung & Rensvold, 2002). Thus, CMB is not a serious concern in study 1.

**Figure 2:**
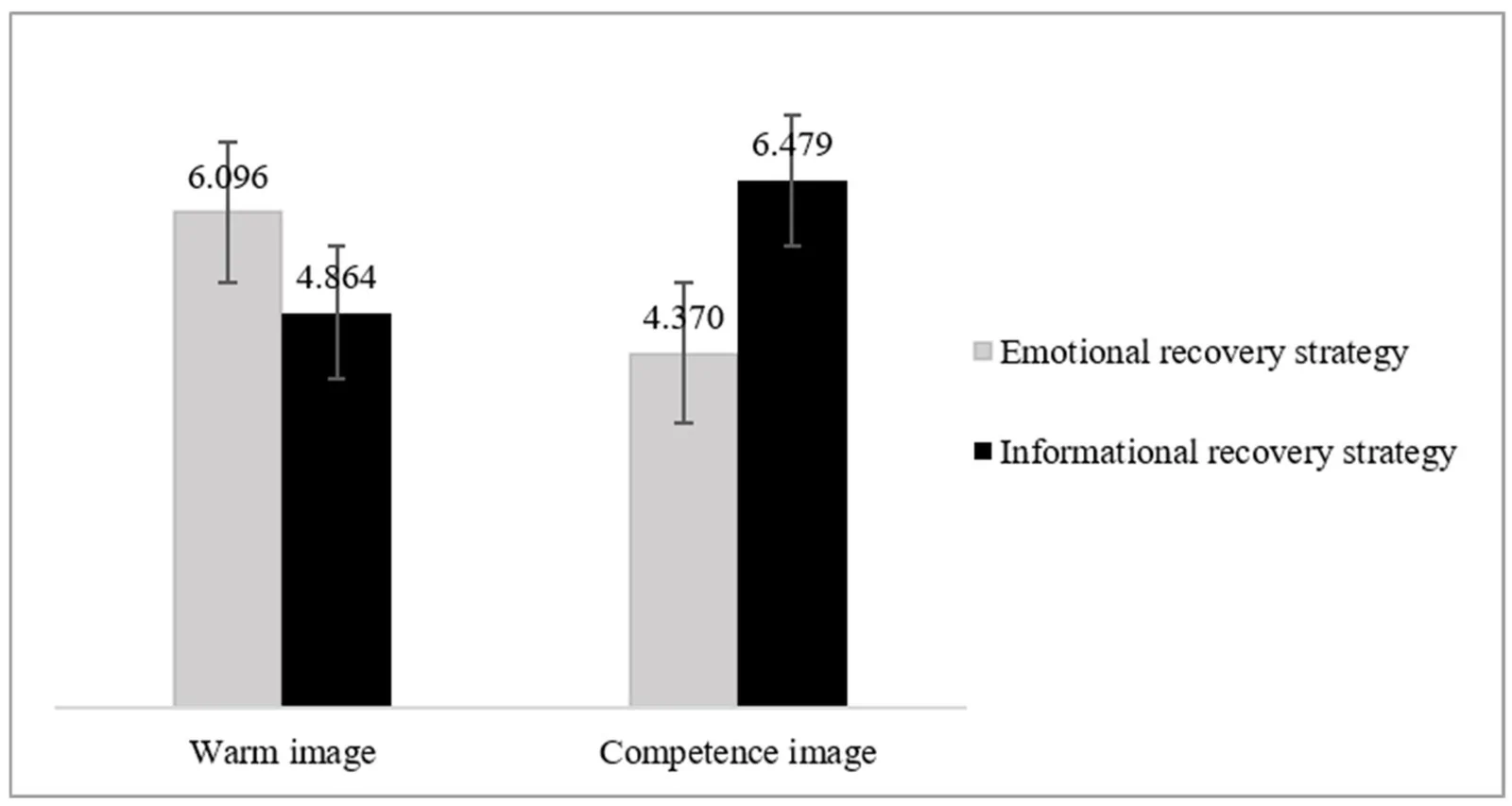
Effects of the interaction of recovery strategies and anthropomorphic image types on recovery satisfaction.

A two-way analysis of covariance (ANCOVA) was conducted to examine the main and interactive effects of recovery strategies and anthropomorphic image types on service recovery satisfaction, with gender, age, monthly disposable allowance, and chatbot usage frequency entered as covariates. The results indicated that the main effect of image type was not significant (F(1, 154) = 0.144, *p* = 0.705), whereas recovery strategy exhibited a significant main effect (F(1, 154) = 9.088, *p* = 0.003). More importantly, the interaction between recovery strategy and image type was significant in influencing service recovery satisfaction (F(1, 154) = 124.384, *p* < 0.001). Among the covariates, gender (F(1, 154) = 1.401, *p* = 0.238), age (F(1, 154) = 0.340, *p* = 0.561), monthly disposable allowance (F(1, 154) = 0.040, *p* = 0.842) and usage frequency (F(1, 154) = 0.714, *p* = 0.4) were all nonsignificant. Overall, these findings provide support for H1. Under the warm image condition, the emotional recovery strategy of chatbots resulted in significantly higher customer satisfaction compared to the informational recovery strategy (M_emotion_ = 6.096 > M_information_ = 4.864, F(1, 154) = 33.978, *p* < 0.001), thereby supporting H1a. In contrast, under the competent image condition, the informational recovery strategy led to significantly higher customer satisfaction than the emotional recovery strategy (M_information_ = 6.479 > M_emotion_ = 4.370, F(1, 154) = 104.813, *p* < 0.001), thereby supporting H1b. Figure 2 and Table 2 further illustrate this interaction effect.

The mediating roles of perceived warmth and perceived competence were assessed using PROCESS Model 8 with 5000 bootstrap resamples. In this analysis, the recovery strategy was specified as the independent variable, perceived warmth and perceived competence as mediators, anthropomorphic image as the moderator, and service recovery satisfaction as the dependent variable. Gender, age, monthly disposable allowance, and usage frequency were included as control variables. The results indicated that none of the control variables were statistically significant.

Table 3 shows the results of the moderated mediation analysis, with statistical significance determined by 95% confidence intervals (CIs) that do not include zero. For the warm image, the chatbot recovery strategy had a significant negative effect on recovery satisfaction (*β* = −0.6256, 95% Boot CI = [−1.0731, −0.178]). Perceived warmth showed a significant indirect effect (*β* = −0.7595, 95% Boot CI = [−1.1204, −0.371]). These results indicate that, under the warm image condition, the emotional recovery strategy leads to higher satisfaction than the informational recovery strategy, supporting H1a. In addition, perceived warmth partially mediates this relationship, supporting H2. For the competent image, the chatbot recovery strategy had a significant positive effect on recovery satisfaction (*β* = 1.9208, 95% Boot CI = [1.5008, 2.3409]). Perceived competence showed a significant indirect effect (*β* = 0.3793, 95% Boot CI = [0.1232, 0.6597]). These results suggest that, under the competent image condition, the informational recovery strategy leads to higher satisfaction than the emotional recovery strategy, supporting H1b. Perceived competence partially mediates this relationship, supporting H3. In addition, the results further show that, under the warm image condition, perceived competence also exerts a significant indirect effect (*β* = 0.1526, 95% Boot CI = [0.0297, 0.3194]).

Furthermore, the moderated mediation effects of perceived warmth (*index* = 0.5682, 95% Boot CI = [0.2147, 1.0212]) and perceived competence (*index* = 0.2267, 95% Boot CI = [0.0489, 0.4869]) were both statistically significant. These results indicate that the mediating effects of perceived warmth and perceived competence differ significantly between the warm image and the competent image.

### 3.2. Study 2: The Moderating Role of Time Pressure

Study 2 was designed to test the moderating effect of time pressure in a controlled laboratory experiment. All participants were recruited from a university located in East China.

#### 3.2.1. Pretest

The pretest was conducted to validate the time pressure manipulation. A total of 56 student participants were randomly allocated to one of the two experimental conditions: low time pressure or high time pressure. Participants were then instructed to read the experimental stimuli. In the low time pressure condition, the scenario was described as follows: imagine that you are traveling to Tianjin for a business trip to attend a conference and deliver a presentation. The meeting is scheduled for 14:00. With two days remaining before the meeting, you need to book your flight and arrange your travel itinerary in advance. You have sufficient time and ample flexibility to plan your trip. The high time pressure scenario was designed as follows. Participants were asked to imagine that they needed to travel to Tianjin for a business trip to attend a meeting and deliver a presentation scheduled at 14:00. At the time of the scenario, it was 08:00 in the morning, leaving only 6 h before the meeting. Participants were required to book a flight immediately and proceed to the airport as soon as possible. They were further informed that any delay in the process could result in missing the meeting.

After reading the scenario, participants completed a measure of perceived time pressure. Time pressure was measured following Lv et al. (2021), using the item: “In this situation, how much available time do you feel you have?” (1 = very sufficient, 7 = very tight), which was used to capture participants’ perceived time pressure (Lv et al., 2021). An independent-samples *t*-test confirmed the effectiveness of the time pressure manipulation (M_low_ = 2.74, SD_low_ = 1.221; M_high_ = 5.52, SD_high_ = 1.149; *t* = −9.629, *p* < 0.001), indicating a successful manipulation.

#### 3.2.2. Experimental Design and Procedure

To improve sample heterogeneity, study 2 recruited student participants from universities located in East China. Given that students from different regions may differ in their exposure to digital service environments and consumption contexts, this sampling strategy helps mitigate potential bias associated with a single-region sample. An a priori power analysis for the between-subjects design was conducted using G*Power 3.1.9 (Faul et al., 2007). The results showed that, with a significance level of 0.05, an effect size of 0.25, and a statistical power of 0.80, the required minimum sample size was 128. This study recruited 356 undergraduate students to participate in the experiment. After screening participants based on their prior chatbot usage experience, 355 eligible participants remained. Following data quality checks, five participants who failed the attention check were removed, yielding a final valid sample of 350 participants for subsequent analysis. The final sample size exceeded the minimum required sample size. Participants were randomly assigned to a 2 (image type: warm image vs. competent image) × 2 (recovery strategy: emotional recovery strategy vs. informational recovery strategy) × 2 (time pressure: low vs. high) between-subjects experimental design. The experimental procedure followed the same structure as study 1, and the manipulations of scenario context, image type, and recovery strategies were consistent with those implemented in study 1.

Study 2 involved offline experimental data collection conducted from April to May 2026 in a behavioral laboratory affiliated with the School of Management at a university located in eastern China. The experimental scenario was presented using a combination of textual and visual stimuli, including the service failure scenario and the manipulations of service recovery strategy, anthropomorphic image, and time pressure. The stimulus materials for the service failure scenario, service recovery strategies, and anthropomorphic images were identical to those used in Study 1, whereas the time pressure manipulation followed the procedure validated in the pretest for Study 2.

During the experiment, participants first completed a screening question to determine whether they had prior experience interacting with chatbots. Those who indicated “No” were excluded from the study, whereas those who indicated “Yes” proceeded to the main questionnaire. The questionnaire was consistent with study 1, with the addition of items measuring perceived time pressure (see Appendix D). Eligible participants were randomly assigned to experimental conditions. They first read a scenario describing time pressure and then viewed stimulus materials illustrating interactions between consumers and chatbots, after which they completed related questions. Participants subsequently completed manipulation checks for time pressure, recovery strategies, and anthropomorphic image. They then reported recovery satisfaction, perceived warmth, and perceived competence, as well as the realism and immersion of the experimental scenario. To ensure data quality, consistent with study 1, an attention check item was embedded in the questionnaire to ensure data quality. Finally, participants reported their demographic information, including gender, age, education, monthly disposable allowance, and frequency of chatbot use. Each participant was compensated with 3 RMB for their participation.

#### 3.2.3. Preliminary Analysis

Manipulation check. A total of 355 participants were collected in this experiment. Five participants failed the attention check and were therefore excluded from the analysis, resulting in a final valid sample of 350 questionnaires. Among the participants, 162 were male (46.3%), and 188 were female (53.7%). The demographic characteristics of the participants are reported in Table 4.

An independent-samples *t*-test indicated successful manipulation of the anthropomorphic image. The warm image was perceived as significantly warmer than the competent image avatar (M_warm_ = 5.797, SD_warm_ = 0.743; M_competent_ = 3.053, SD_competent_ = 0.818; *t* = 32.841, *p* < 0.001). Conversely, the competent image was perceived as significantly more competent than the warm image (M_competent_ = 6.034, SD_competent_ = 0.617; M_warm_ = 3.386, SD_warm_ = 1.004; *t* = −29.740, *p* < 0.001). The manipulation checks further confirmed the effectiveness of the experimental conditions. The chatbot recovery strategy manipulation was successful (M_emotion_ = 2.96, SD_emotion_ = 1.618; M_information_ = 5.86, SD_information_ = 1.268; *t* = −18.691, *p* < 0.001), as was the time pressure manipulation (M_low_ = 3.31, SD_low_ = 1.626; M_high_ = 5.80, SD_high_ = 1.194; *t* = −16.407, *p* < 0.001).

Moreover, a one-sample *t*-test showed that participants rated both scenario realism (M = 5.930, SD = 0.557; *t* = 199.264, *p* < 0.001) and scenario immersion (M = 6.062, SD = 0.637; *t* = 178.146, *p* < 0.001) as high, indicating that the experimental settings were perceived as realistic and immersive.

Assessment of measurement. The measurement scales were identical to those employed in study 1. All constructs were assessed using a seven-point Likert scale ranging from 1 = strongly disagree to 7 = strongly agree. The Cronbach’s alpha values indicated good internal consistency, with recovery satisfaction (Cronbach’s α = 0.887), perceived warmth (Cronbach’s α = 0.807), perceived competence (Cronbach’s α = 0.872), scenario realism (Cronbach’s α = 0.799), and scenario immersion (Cronbach’s α = 0.831). Appendix C reports Cronbach’s α, composite reliability and average variance extracted for all constructs.

#### 3.2.4. Results

A total of 350 participants who passed the attention check were included in the data analysis. To mitigate evaluation anxiety and reduce potential self-report bias, all responses were collected anonymously (Podsakoff et al., 2012). Harman’s single-factor test was conducted to examine common method bias in the dataset. The results indicated that a single factor explained 26.052% of the total variance, less than the 50% baseline (Podsakoff et al., 2012). Furthermore, we adopted the unmeasured latent method construct (ULMC) approach. The baseline model demonstrated reasonable fit (χ^2^/df = 2.909, CFI = 0.928, RMSEA = 0.074). Following the introduction of the common latent factor (CLF), the model fit indices remained largely unchanged (χ^2^/df = 3.108, CFI = 0.933, RMSEA = 0.078), and the change in CFI (ΔCFI = 0.005) remained below the 0.01 threshold suggested (Cheung & Rensvold, 2002). Thus, CMB is not a serious concern in study 2.

A three-way ANCOVA was conducted with service recovery satisfaction as the dependent variable. The results indicated a significant interaction effect among recovery strategy, image type, and time pressure (F(1, 338) = 4.527, *p* = 0.034). Regarding the covariates, gender (F(1, 338) = 0.016, *p* = 0.9), age (F(1, 338) = 0.332, *p* = 0.565), monthly disposable allowance (F(1, 338) = 0.365, *p* = 0.546), and usage frequency (F(1, 338) = 0.46, *p* = 0.498) were not significant. Pairwise comparisons and simple effect analyses (see Table 5) showed that under high time pressure, a competent image combined with an informational recovery strategy (compared with a warm image combined with an emotional recovery strategy) led to higher customer recovery satisfaction, thereby supporting H4. Under low time pressure, a warm image combined with an emotional recovery strategy (compared with a competent image combined with an informational recovery strategy) resulted in higher customer recovery satisfaction, thereby supporting H5.

## 4. Discussion

### 4.1. Research Findings

Prior research has primarily focused on the effects of a single social cue of chatbots on consumer responses, whereas how multiple social cues jointly shape consumers’ social cognition, psychological evaluations, and ultimately service recovery outcomes remains insufficiently explored. Accordingly, this study investigates how the match between chatbots’ linguistic cues (recovery strategies) and visual cues (anthropomorphic images) affects service recovery satisfaction.

The results indicate that there is a matching effect between chatbots’ recovery strategies and anthropomorphic image types, such that the congruence between linguistic cues and visual cues enhances consumers’ service recovery satisfaction (Study 1, supporting H1). Specifically, consumers reported higher service recovery satisfaction when a chatbot’s warm image was matched with an emotional recovery strategy (Study 1, supporting H1a). Likewise, a chatbot’s competent image matched with an informational recovery strategy led to higher service recovery satisfaction (Study 1, supporting H1b).

Further analysis indicates that, based on the Stereotype Content Model (SCM), perceived warmth and perceived competence play mediating roles (Study 1). The match between the emotional recovery strategy and a warm image can enhance consumers’ psychological perceptions of kindness toward the chatbot, namely perceived warmth, thereby increasing service recovery satisfaction (Study 1, supporting H2). In contrast, the alignment between the informational recovery strategy and a competent image can strengthen consumers’ psychological evaluation of the chatbot’s problem-solving ability, namely perceived competence, thereby increasing service recovery satisfaction (Study 1, supporting H3). Moreover, this study unexpectedly revealed that perceived competence also mediated the positive effect of the congruence between an emotional recovery strategy and a warm image on service recovery satisfaction. A possible explanation is that, in service recovery contexts, an emotional recovery strategy not only provides emotional support but also communicates that the chatbot understands consumers’ concerns, with such understanding serving as a competence-related cue. When an emotional recovery strategy is combined with a warm image, the warm image may enhance consumers’ acceptance of this competence cue, thereby activating perceptions of the chatbot’s competence and subsequently improving service recovery satisfaction (S. Wang et al., 2023). This mechanism is distinct from the mediating role of perceived warmth, indicating that the congruence between a warm image and an emotional recovery strategy simultaneously enhances service recovery satisfaction through two independent psychological pathways: perceived warmth and perceived competence.

Finally, time pressure moderates the interactive effect of recovery strategies and anthropomorphic image on service recovery satisfaction (Study 2). Under high time pressure, consumers tend to prioritize efficiency and information accuracy in interactions with chatbots (Q. N. Nguyen et al., 2022). Accordingly, the congruence between informational recovery strategies and a competent image leads to higher service recovery satisfaction (Study 2, supporting H4). In contrast, under low time pressure, consumers place greater emphasis on the emotional experience of interacting with chatbots (Y. S. S. Huang & Dootson, 2022). Therefore, the congruence between emotional recovery strategies and a warm image enhances service recovery satisfaction (Study 2, supporting H5).

### 4.2. Theoretical Contributions

This study makes important contributions to the literature on chatbot service recovery. First, this study extends the application of the CASA paradigm in chatbot service recovery contexts. Existing research on chatbot service recovery grounded in the CASA paradigm has primarily examined the effects of individual social cues on consumer responses, including linguistic cues such as emotional expressions (Zhang et al., 2025) and social mindfulness (H. Meng et al., 2025b), as well as visual cues such as anthropomorphic appearances (Pavone et al., 2023; Agnihotri & Bhattacharya, 2024). This study demonstrates how the integration of two types of social cues jointly shapes consumers’ service recovery evaluations. Furthermore, it provides a theoretical explanation for the inconsistent findings regarding the effects of anthropomorphism in existing research. Prior studies have suggested that anthropomorphism may facilitate positive consumer responses in service recovery contexts (Agnihotri & Bhattacharya, 2024; H. Liu et al., 2026), while other studies have documented its potential negative effects (Esmark Jones et al., 2026). This study further indicates that these inconsistent findings may partly stem from previous research overlooking the matching relationship between anthropomorphic image types and recovery strategies. By highlighting the importance of such congruence, this study advances the understanding of why anthropomorphism produces different effects across service recovery contexts. Specifically, the findings show that consumers report higher service recovery satisfaction when an emotional recovery strategy is congruent with a warm image and when an informational recovery strategy is congruent with a competent image. These findings extend the CASA paradigm beyond the effects of individual social cues by demonstrating how multiple social cues jointly operate in chatbot interactions. This study further advances the understanding of social cue interaction mechanisms within the CASA framework and contributes to the broader human–computer interaction literature (Wu et al., 2024, 2025b; C. Liu et al., 2026).

Second, this study confirms the applicability of warmth and competence as fundamental dimensions of social cognition in chatbot service recovery contexts, thereby reinforcing the theoretical relevance of the SCM in explaining how consumers form social evaluations of artificial intelligence agents. Specifically, during chatbot service recovery, the interaction between recovery strategies and anthropomorphic image types influences consumers’ perceived warmth and perceived competence, which reflect consumers’ psychological judgments of chatbots based on integrated social cues and further explain how consumers’ interpretations of these social cues influence service recovery satisfaction. Furthermore, the unexpected findings of this study provide additional insights into the application of the SCM in chatbot service recovery contexts. This study further reveals that the congruence between an emotional recovery strategy and a warm image not only enhances consumers’ perceived warmth but also strengthens their perceived competence. This finding suggests that, in chatbot service recovery contexts, consumers’ social cognition is not confined to the specific cognitive dimension directly associated with a given social cue. Instead, consumers integrate social information from multiple sources and form broader, cross-dimensional evaluations of chatbots. This finding extends prior understanding of the relationship between social cues and their corresponding cognitive dimensions by demonstrating the interconnectedness among different dimensions of social cognition. It further enriches the explanatory scope of the SCM in the context of artificial intelligence-based social interactions.

Finally, this study identifies time pressure as an important boundary condition that moderates the matching effect between recovery strategies and anthropomorphic image types. Previous research has mainly examined the role of time pressure in shaping the effectiveness of emotional recovery strategies (Song et al., 2023; H. Meng et al., 2025b; Juquelier et al., 2025), with limited attention to its role in influencing the effectiveness of recovery strategies and anthropomorphic image type congruence. Specifically, under low time pressure conditions, the alignment between an emotional recovery strategy and a warm image is more likely to enhance service recovery satisfaction. In contrast, under high time pressure conditions, the alignment between an informational recovery strategy and a competent image is more effective in improving service recovery satisfaction. These findings indicate that the effectiveness of recovery strategy and anthropomorphic image type congruence varies depending on contextual conditions, thereby extending the understanding of the boundary conditions of social cue congruence effects in chatbot service recovery.

### 4.3. Practical Implications

The findings of this study have practical implications for firms and chatbot service providers. First, service providers should prioritise the design of chatbots’ self-recovery. Compared with human-agent recovery, chatbots’ self-recovery is more cost-efficient and can operate continuously, which accounts for their widespread use in service failure situations (Ashfaq et al., 2020; Bagherzadeh et al., 2020; Brendel et al., 2023; D. Liu et al., 2023). Meanwhile, existing research suggests that, against the backdrop of ongoing digital-intelligence transformation, users’ behavioural responses to intelligent systems, as well as their evaluation processes, are significantly shaped (W. B. Kim & Hur, 2024; Wu et al., 2025a). To enhance user experience, recovery strategies should match the type of chatbot’s anthropomorphic image: when the chatbot displays a warm image, an emotional strategy can enhance user satisfaction; when it displays a competent image, an informational strategy can improve service recovery effectiveness. By implementing this matching mechanism, firms can optimize the design of chatbot recovery strategies in service failure contexts, thereby enhancing users’ service experience.

Second, research indicates that perceived warmth and perceived competence positively influence service recovery satisfaction, providing new practical guidance for enhancing recovery outcomes. When a chatbot presents a warm image, its emotional strategy can more effectively increase users’ perceived warmth. Therefore, when designing emotional strategies, service providers should focus on elements that meet customers’ emotional needs, such as empathy, care, and apologies. When a chatbot presents a competent image, its informational strategy can significantly enhance users’ perceived competence. Firms can continuously improve informational strategies using machine learning and dynamic dialogue optimisation, emphasising the explanation of problem causes, the provision of feasible solutions, and additional relevant information, thereby enhancing users’ perceptions of the chatbot’s problem-solving capabilities. In practice, these measures can help firms carry out recovery quickly and efficiently after service failures, enhancing customers’ service recovery satisfaction.

When firms design matching strategies for chatbot recovery strategies and anthropomorphic image types, they should also consider customers’ time pressure to improve service recovery effectiveness. Specifically, firms can use predefined systems to enable chatbots to remain sensitive to time-related information, thereby appropriately adjusting recovery strategies. For example, firms can identify customers’ time pressure based on message length, the urgency reflected in their tone, and whether they greet the chatbot. When consumers are under low time pressure, firms can match an emotional recovery strategy with a warm image to achieve more effective service recovery. In contrast, when consumers are under high time pressure, firms can match an informational recovery strategy with a competent image to enhance service recovery satisfaction. Therefore, chatbots should optimize the combination of strategies and image types under different levels of time pressure to enhance service recovery effectiveness and customer experience.

### 4.4. Limitations and Future Research

Although this study extends the literature on chatbot service recovery, several limitations remain that warrant further investigation. First, this study focuses on two types of anthropomorphic images, namely warm and competent images. Future research could extend this framework to other dimensions, such as chatbot role and gender. Prior studies have shown that chatbot roles (servant or companion) significantly influence users’ continuance intention (Shen et al., 2025). Moreover, in service failure contexts, the role positioning of service robots has been found to affect consumers’ dysfunctional behaviors (B. Huang et al., 2025; Lei & Xie, 2025). Future research could further examine the interaction effects between chatbot recovery strategies and role positioning on service recovery satisfaction in service failure contexts. In addition, prior studies have investigated the impact of chatbot gender on recovery satisfaction in service failure situations (Liang et al., 2024; Rese & Witthohn, 2025). Future research could extend this line of inquiry by examining the interaction effects between recovery strategies and gender.

Second, this study examines the moderating role of time pressure. Future research could extend this work by investigating other potential boundary conditions that may shape the effectiveness of chatbot service recovery. Prior studies have demonstrated that factors such as failure attribution (Castillo et al., 2024) and task type (Chen, 2024) significantly influence service recovery satisfaction. Moreover, future research could further examine chatbot service recovery effectiveness across different levels of failure severity (Shams et al., 2024; C. Wang et al., 2025) and failure types (Xing et al., 2022), thereby providing a more comprehensive understanding of the boundary conditions of chatbot service recovery effectiveness.

Third, this study employed an offline laboratory experiment using a sample of Chinese university students. Although laboratory experiments provide strong control over the research context, they may also limit the ecological validity of the findings. Future research could adopt online experiments, field studies, or other methodological approaches to further examine the robustness of the findings. Moreover, while student samples typically have relatively high levels of digital technology experience, their demographic characteristics, including age, occupation, and educational background, tend to be relatively homogeneous, which may constrain the external validity of the findings. Future research can include consumer samples with diverse ages, occupations, and educational backgrounds and extend the research context to different cultural settings to examine the applicability of the research model across different groups and cultural environments, thereby further improving the generalizability and robustness of the findings.

## Figures and Tables

**Table 1:** Descriptive statistics of respondents in Study 1.

Respondents	Category	Count	Proportion
Gender	Male	63	38.9%
	Female	99	61.1%
Age	Under 20	72	44.4%
	21–30	90	55.6%
Education	College/University	162	100%
Monthly disposable allowance	CNY 1000–2000	85	52.5%
	CNY 2000–3000	67	41.4%
	More than CNY 3000	10	6.2%
Frequency of chatbot usage	Seldom	2	1.2%
	Sometimes	15	9.3%
	Often	58	35.8%
	Always	87	53.7%

**Table 2:** Pairwise comparisons of recovery satisfaction.

Anthropomorphic Image Type	Comparison	Mean	Mean Difference	SE	Sig.
Warm image	Emotional recovery strategy	6.096	1.232	0.211	<0.001
	Informational recovery strategy	4.864			
Competence image	Emotional recovery strategy	4.370	−2.109	0.206	<0.001
	Informational recovery strategy	6.479			

**Table 3:** Moderated mediation effect in study 1.

Moderator	Direct Effect	*p*	95%Boot CI		Mediator	Indirect Effects	95%Boot CI	
			LLCI	ULCI			LLCI	ULCI
Warm image	−0.6256	0.0065	−1.0731	−0.178	Perceived warmth	−0.7595	−1.1204	−0.371
Competent image	1.9208	<0.001	1.5008	2.3409		−0.1913	−0.4141	0.257
Index of moderated mediation						0.5682	0.2147	1.0212
Warm image	−0.6256	0.0065	−1.0731	−0.178	Perceived competence	0.1526	0.0297	0.3194
Competent image	1.9208	<0.001	1.5008	2.3409		0.3793	0.1232	0.6597
Index of moderated mediation						0.2267	0.0489	0.4869

**Table 4:** Descriptive statistics of respondents in Study 2.

Respondents	Category	Count	Proportion
Gender	Male	162	46.3%
	Female	188	53.7%
Age	Under 20	165	47.1%
	21–30	185	52.9%
Education	College/University	350	100%
Monthly disposable allowance	CNY 1000–2000	58	16.6%
	CNY 2000–3000	226	64.6%
	More than CNY 3000	66	18.9%
Frequency of chatbot usage	Sometimes	10	2.9%
	Often	126	36%
	Always	214	61.1%

**Table 5:** Pairwise comparisons (recovery strategy × anthropomorphic image type × time pressure) of recovery satisfaction.

Image	Strategy	Time Pressure	Mean	Mean Difference	SE	F	Sig.
Warm image	Emotion	Low	5.097	0.445	0.218	F(1, 338) = 4.151	0.042
		High	4.652				
Warm image	Information	Low	4.588	0.082	0.222	F(1, 338) = 0.135	0.714
		High	4.506				
Competent image	Emotion	Low	4.523	0.381	0.219	F(1, 338) = 3.015	0.083
		High	4.142				
Competent image	Information	Low	4.479	−0.922	0.224	F(1, 338) = 16.958	<0.001
		High	5.401				

## Data Availability

The raw data supporting the conclusions of this article will be made available by the authors on request. The data are not publicly available due to privacy considerations.

## Appendix A. Experimental Material in Study 1 and Study 2

1. Emotional recovery strategy/Informational recovery strategy in Warm image
  	Emotional Recovery Strategy	Informational Recovery Strategy
  Warm image		
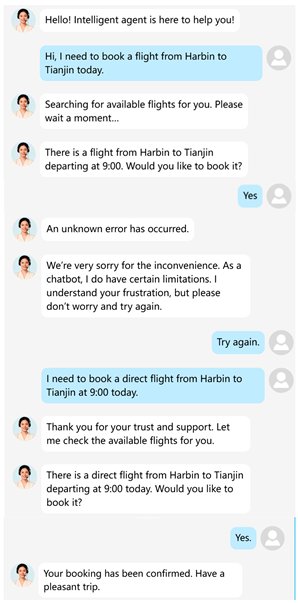
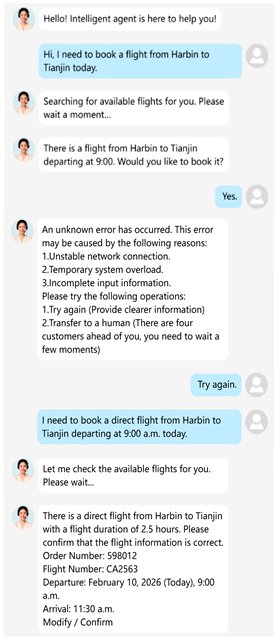

2. Emotional recovery strategy/Informational recovery strategy in Competence image
  	Emotional Recovery Strategy	Informational Recovery Strategy
  Competence image		
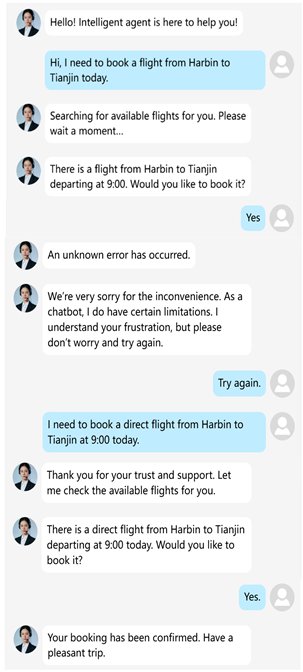
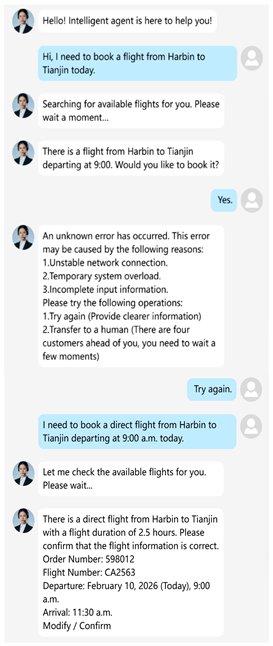

## Appendix B. Measurement in Study 1 and Study 2

**Construct**	**Items**	**References**
Recovery Satisfaction	I am satisfied with the chatbot.	(Chung et al., 2020)
	The chatbot did a good job.	
	The chatbot met my expectations.	
	The conversation with the chatbot was satisfactory.	
Perceived Warmth	I think the chatbot is friendly.	(Gao & Mattila, 2014)
	The chatbot makes me feel encouraged.	
	I feel that the chatbot shows empathy toward me.	
Perceived Competence	I find the chatbot competent.	(Cuddy et al., 2008)
	I find the chatbot efficient.	
	I find the chatbot skilled.	
Scenario Realism	This scenario is closely aligned with real-life situations.	(Crolic et al., 2022)
	The scenario is realistic.	
	It is easy to imagine being in the situation described in this study.	
Scenario Immersion	I can imagine myself as the main character of the above situation.	(Song et al., 2022)
	It’s easy for me to imagine myself in this service scenario.	
	I can resonate with the emotions and experiences of the characters in the mentioned scenario.	

## Appendix C. Full Scale Items

**Construct**	**Study 1**			**Study 2**		
	**α**	**CR**	**AVE**	**α**	**CR**	**AVE**
Recovery Satisfaction	0.940	0.940	0.797	0.887	0.887	0.663
Perceived Warmth	0.905	0.906	0.762	0.807	0.807	0.583
Perceived Competence	0.828	0.828	0.616	0.872	0.873	0.696
Scenario Realism	0.840	0.843	0.642	0.799	0.805	0.583
Scenario Immersion	0.770	0.777	0.541	0.831	0.833	0.625
Warm image	0.944	0.945	0.811	0.931	0.934	0.782
Competent image	0.959	0.959	0.853	0.937	0.938	0.790

## Appendix D. Questionnaire in Study 1 and Study 2

Have you used a chatbot before?
  □ Yes
  □ No

Warm image [1 = Strongly Disagree, 7 = Strongly Agree]
  □ Enthusiastic
  □ Friendly
  □ Warm
  □ Sincere

Competence image [1 = Strongly Disagree, 7 = Strongly Agree]
  □ Competent
  □ Smart
  □ Efficient
  □ Skilled

The chatbot’s recovery strategy is more inclined toward an emotional recovery strategy or an informational recovery strategy [1 = Strongly Disagree, 7 = Strongly Agree]
  □ Emotional recovery strategy
  □ Informational recovery strategy

In this situation, how much available time do you feel you have? [1 = Very sufficient, 7 = Very tight] [Study 2]
  □ Very sufficient
  □ Very tight

Scenario Realism [1 = Strongly Disagree, 7 = Strongly Agree]
  □ This scenario is closely aligned with real-life situations.
  □ The scenario is realistic.
  □ It is easy to imagine being in the situation described in this study.

Scenario Immersion [1 = Strongly Disagree, 7 = Strongly Agree]
  □ I can imagine myself as the main character of the above situation.
  □ It’s easy for me to imagine myself in this service scenario.
  □ I can resonate with the emotions and experiences of the characters in the mentioned scenario.

Recovery Satisfaction [1 = Strongly Disagree, 7 = Strongly Agree]
  □ I am satisfied with the chatbot.
  □ The chatbot did a good job.
  □ The chatbot met my expectations.
  □ The conversation with the chatbot was satisfactory.

Perceived Warmth [1 = Strongly Disagree, 7 = Strongly Agree]
  □ I think the chatbot is friendly.
  □ The chatbot makes me feel encouraged.
  □ I feel that the chatbot shows empathy toward me.

Perceived Competence [1 = Strongly Disagree, 7 = Strongly Agree]
  □ I find the chatbot competent.
  □ I find the chatbot efficient.
  □ I find the chatbot skilled.

Attention check: Please select [Strongly Disagree] for this question
  □ Strongly Disagree
  □ Disagree
  □ Agree
  □ Strongly Agree

What’s your gender?
  □ Male
  □ Female

How old are you?
  □ <20
  □ 21–30

What is your highest level of education?
  □ High school or below
  □ Associate degree
  □ Undergraduate degree
  □ Postgraduate degree

What is your average monthly disposable income (including parental support, part-time earnings, scholarships, and other sources)?
  □ Less than CNY 1000
  □ CNY 1000–2000
  □ CNY 2000–3000
  □ More than CNY 3000

How often do you use chatbots?
  □ Never
  □ Seldom
  □ Sometimes
  □ Often
  □ Always
